# Community detection in hypergraphs via mutual information maximization

**DOI:** 10.1038/s41598-024-55934-5

**Published:** 2024-03-23

**Authors:** Jürgen Kritschgau, Daniel Kaiser, Oliver Alvarado Rodriguez, Ilya Amburg, Jessalyn Bolkema, Thomas Grubb, Fangfei Lan, Sepideh Maleki, Phil Chodrow, Bill Kay

**Affiliations:** 1 Department of Mathematical Sciences, Carnegie Mellon University, Pittsburgh, PA 15213 USA; 2 Department of Computer Science, New Jersey Institute of Technology, Newark, NJ 07102 USA; 3 Pacific Northwest National Laboratory, Richland, WA 99354 USA; 4 Department of Mathematics, California State University, Dominguez Hills, Carson, CA 90747 USA; 5 University of California San Diego, San Diego, CA 92093 USA; 6 Department of Informatics, Indiana University, Bloomington, IN 47408 USA; 7 Scientific Computing and Imaging Institute, University of Utah, Salt Lake City, UT 84112 USA; 8 Department of Computer Science, University of Texas at Austin, Austin, TX 78712 USA; 9 Department of Computer Science, Middlebury College, Middlebury, VT 05753 USA

**Keywords:** Mathematics and computing, Computational science, Scientific data

## Abstract

The hypergraph community detection problem seeks to identify groups of related vertices in hypergraph data. We propose an information-theoretic hypergraph community detection algorithm which compresses the observed data in terms of community labels and community-edge intersections. This algorithm can also be viewed as maximum-likelihood inference in a degree-corrected microcanonical stochastic blockmodel. We perform the compression/inference step via simulated annealing. Unlike several recent algorithms based on canonical models, our microcanonical algorithm does not require inference of statistical parameters such as vertex degrees or pairwise group connection rates. Through synthetic experiments, we find that our algorithm succeeds down to recently-conjectured thresholds for sparse random hypergraphs. We also find competitive performance in cluster recovery tasks on several hypergraph data sets.

## Introduction

The network clustering task asks us to identify sets, or *clusters*, of related vertices in a network. In particular, we aim to identify groups of vertices that are related to each other in some way that they are not related to vertices in other clusters. In various disciplines, the graph clustering task may also be called *network partitioning* or *community detection*. A large number of methods have been developed for clustering dyadic networks, in which relationships exist between pairs of vertices. Such dyadic networks can be represented as graphs. Techniques for graph clustering include spectral methods, greedy optimization methods, and methods based on statistical inference, with many theoretical connections across these categories^1^.

Much recent work has emphasized the importance of multiway relations—interactions between groups of two or more entities—in complex systems^2,3^. Such interactions can often be modeled as edges in a generalization of graphs usually referred to as hypergraphs. A *hypergraph* $\textsf{H}=(V, E)$ consists of a finite set of vertices *V* and a collection of edges $E \subseteq \mathscr {P}(V)$ (the power set of the vertex set). That is, hypergraphs generalize graphs by allowing edge sizes other than two. Hypergraphs pose both opportunities and challenges for clustering algorithms. On the one hand, the richer representation of relationships offered by hypergraphs can in some cases produce superior performance when compared to graph methods applied to the same data. On the other hand, the flexibility implied by arbitrary edge sizes can lead to both computational and statistical pitfalls. There are many extant approaches to hypergraph clustering including spectral methods^4^, methods based on combinatorial optimization^5–7^, and methods based on statistical inference in both single-membership and mixed-membership generative models^8,9^.

In this paper, we offer a hypergraph clustering algorithm with information-theoretic foundations. This algorithm extends a method proposed by Rosvall and Bergstrom for graph clustering^10^. Their approach begins by regarding a proposed clustering of a graph as a lossy *compression* of the graph. The goal, then, is to form a compression that, for a fixed storage size, is maximally informative of the original graph structure. They formulate this criterion in terms of maximization of mutual information, or, equivalently, minimization of a certain entropy functional. They then use simulated annealing to perform the minimization. This approach is equivalent to maximum-likelihood estimation in a microcanonical graph stochastic blockmodel^11^, and may thus also be viewed as a statistical inference method.

We note here that there are a number of different notions of “community” that depend on context. In reference to real world data, communities are observed labels of the data points (for example, classroom assignments for students). If a graph is generated by sampling from a stochastic blockmodel, then the communities reference the latent partitions that are a parameter of the stochastic blockmodel. In our information-theoretic approach, communities are a partition of the vertex set of a graph that is used to compress the graph. There are two heuristics at play. First, there is an assumption that observed edges in real world data are informative of the communities (data point label), in much the same way that edges in the stochastic blockmodel are more likely to be inside of communities (as a parameter of the stochastic blockmodel) under certain parameter choices. Second, we assume that communities (as a partition) for compression should co-vary with communities (as a parameter) in graphs sampled from the stochastic blockmodel. Our approach is to estimate the communities for compression as a way to approximate communities for a stochastic blockmodel or ground truth labels in real world data sets.

The main contribution of this paper is to extend the algorithm of Rosvall-Bergstrom to hypergraphs by (a) formulating the entropy functional on the more combinatorially complex set of hypergraphs and (b) incorporating a *degree-correction*^11,12^ to account for heterogeneity of vertex degrees. Our algorithm is native to the hypergraph, but reduces to the graph version on 2-uniform hypergraphs. Section “Methods” contains a description of the entropy functional, its information theoretic foundations, the simulated annealing algorithm we use to locally minimize the entropy, and a principled method for determining a target number of clusters. In Section “Results: synthetic data”, we demonstrate our algorithm on several synthetic data sets, finding experimental suggestion that the algorithm succeeds down to the sparse detectability limit conjectured by Chodrow et al. ^8^ In Section “Results: experiments on data”, we conduct experiments on several empirical data sets, finding performance competitive with extant graph and hypergraph methods. We close in Section “Discussion” with discussion of our findings and suggestions for future work.

## Methods

We treat the hypergraph clustering problem as an information-theoretic compression problem in which the aim is to find a maximally informative clustered description of the hypergraph structure. In this section, we introduce the core technical ideas needed to describe this approach: hypergraph compressions, information, and entropy.

### Hypergraph compression

Let $\textsf{H}$ be a hypergraph with edge set $E=E(\textsf{H})$ and vertex set $V=V(\textsf{H})$. Suppose $\left\{ C_i\right\} _{i=1}^m$ is a partition of *V* into *m* clusters. For $\lambda = (\lambda _1, \ldots , \lambda _m)\in \mathbb N^{m}$, we say an *edge* $A\in E$ *is of* $\lambda$-*type* if $\left| A\cap C_i\right| =\lambda _i$ for $1\le i \le m$. That is, $\lambda _i$ counts the number of vertices in edge *A* belonging to cluster $C_i$. We denote by $E_\lambda$ the set of all edges of $\lambda$-type.

#### Definition 2.1

*(Hypergraph Compression)* A *compression of* $\textsf{H}$ *into* *m* *clusters* is a pair $\gamma = (\left\{ C_i\right\} _{i=1}^m,\left\{ e_\lambda \right\} _{\lambda \in \mathbb N^{m}})$ such that

- $\left\{ C_i\right\} _{i=1}^m$ is a partition of *V*, and
- $\left\{ e_\lambda \right\} _{\lambda \in \mathbb N^{m}}$ is a collection indexed by $\lambda$, where $e_\lambda$ is the number of $\lambda$-type edges in $\textsf{H}$.

We say that $\textsf{H}$ and $\gamma$ are *compatible* if $\gamma$ is a compression of $\textsf{H}$. We let $\mathbb {H}(\gamma )$ be the set of all hypergraphs compatible with a fixed $\gamma$, and let $Z(\gamma ) = \left| \mathbb {H}(\gamma )\right|$. We also let $\Gamma (\textsf{H})$ denote the set of compressions compatible with $\textsf{H}$.

The collection of clusters $\left\{ C_i\right\} _{i=1}^m$ may be equivalently represented as an assignment vector ${\textbf {c}}\in \left\{ 1,\dots ,m\right\} ^{V}$ where $c_v=i$ if and only if $v\in C_i$. Similarly, if $\textsf{H}$ is a simple graph, then $\left\{ e_\lambda \right\} _{\lambda \in \mathbb N^{m}}$ reduces to the module matrix in simple graph formulations of compression^10^.

In applications, it is useful to also incorporate the vertex degree sequence into the compressed representation of the hypergraph. Let $\left\{ d_v\right\} _{v\in V}$ be the degree sequence of vertices in $\textsf{H}$.

#### Definition 2.2

*(Degree-Corrected Hypergraph Compression)* A *compression of* $\textsf{H}$ *into* *m* *clusters with degrees* is a triple $\gamma = (\left\{ C_i\right\} _{i=1}^m,\left\{ e_\lambda \right\} _{\lambda \in \mathbb N^{m}},\left\{ d_v\right\} _{v\in V})$.

**Figure 1:**
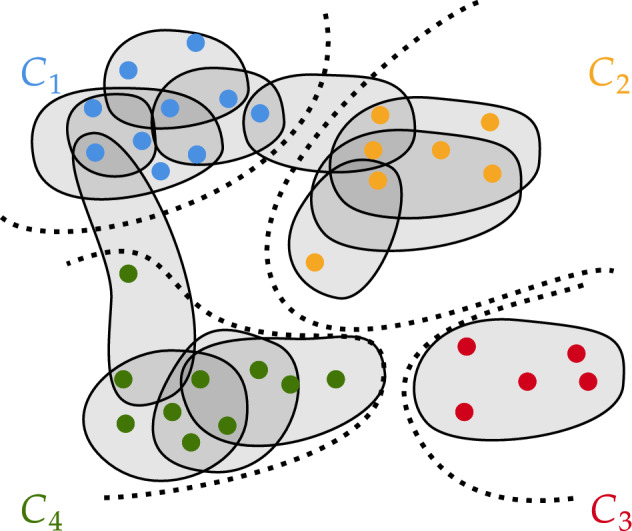
Example of a hypergraph whose vertices have been partitioned into four clusters.

Explicitly incorporating the degree sequence into the compression is the analogue of degree-correction in canonical stochastic blockmodels^12^. In Section “Results: experiments on data”, we will see that the degree-corrected compressions give improved ARI (Adjusted Rand Index) when clustering against known ground truths. This finding is consistent with simple graph clustering ^13^, which typically have heterogeneous degree sequences. We discuss the connection to stochastic blockmodels in Section “Relation to maximum-likelihood estimation”. Throughout the remainder of this paper, we let $\Gamma (\textsf{H})$ denote the set of all compressions of a fixed hypergraph $\textsf{H}$, describing in context when necessary whether the space of compressions includes degrees. An example of a clustered hypergraph can be found in Fig. 1.

### Information and entropy

For a given hypergraph, our aim is to select a maximally informative compression. We define the information content of a compression in terms of Shannon entropy^14^. Our definitions follow the formulation of Cover and Thomas^15^ Let *X* and *Y* be discrete random variables with joint distribution *p*(*x*, *y*) over an alphabet $\mathscr {X}\times \mathscr {Y}$.

#### Definition 2.3

*(Marginal, Joint, and Conditional Entropies)* The *marginal entropy* (or simply *entropy*) of the random variable *X* is

$$\begin{aligned} H(X) \triangleq - \sum _{x \in \mathscr {X}}p(x) \log p(x)\;. \end{aligned}$$

The *joint entropy* of *X* and *Y* is

$$\begin{aligned} H(X, Y) \triangleq - \sum _{x \in \mathscr {X}}\sum _{y \in \mathscr {Y}} p(x, y) \log p(x, y)\;. \end{aligned}$$

The *conditional entropy* of *Y* given *X* is

$$\begin{aligned} H(Y|X) = - \sum _{x \in \mathscr {X}}\sum _{y \in \mathscr {Y}} p(x, y) \log p(y|x)\;. \end{aligned}$$

The entropy *H*(*X*) can be viewed as a measure of spread for the discrete random variable *X*. It is maximized with respect to the distribution *p* by the uniform distribution $p(x) = \frac{1}{\left| \mathscr {X}\right| }$, in which case $H(X) = \log \left| \mathscr {X}\right|$. The joint entropy *H*(*X*, *Y*) is similarly a measure of spread for the joint distribution *p*(*X*, *Y*). The conditional entropy *H*(*Y*|*X*) is the expected spread in the distribution *p*(*y*|*x*) across realizations of *x*, as highlighted by the formula

$$\begin{aligned} H(Y|X)&= - \sum _{x \in \mathscr {X}}\sum _{y \in \mathscr {Y}} p(x)p(y|x) \log p(y|x) \\&= -\sum _{x \in \mathscr {X}}p(x)H(Y|X = x)\;. \end{aligned}$$

#### Definition 2.4

*(Mutual Information)* The *mutual information* of *X* and *Y* is given by:

$$\begin{aligned} I(X;Y)&\triangleq H(X) - H(X|Y)\\&=H(Y) - H(Y|X)\;. \end{aligned}$$

Other definitions of the mutual information exist under which Definition 2.4 is a theorem rather than a definition. Treating *H*(*X*) as a measure of uncertainty about *X*, and *H*(*X*|*Y*) as a measure of uncertainty about *X* conditional on knowing the value of *Y*, the mutual information measures how much knowledge of *Y* *reduces* uncertainty in *X*.

### Information maximization as counting

Our aim is to choose a compression $\gamma$ that is maximally informative about the structure of the hypergraph $\textsf{H}$. Let $\Gamma$ be a set of possible compressions and, for each $\gamma \in \Gamma$, $p(\cdot ~|~\gamma )$ be uniform on $\mathbb {H}(\gamma )$. In practice, we usually take $\Gamma = \Gamma (\textsf{H}_0)$ to be the set of all compressions compatible with an observed hypergraph $\textsf{H}_0$. We assume an unspecified prior *q* over $\Gamma$ which we will soon optimize. We model $\textsf{H}$ as being drawn from a distribution:

$$\begin{aligned} p(\textsf{H}) = \sum _{\gamma \in \Gamma } p(\textsf{H}~|~ \gamma )q(\gamma )\;. \end{aligned}$$

We form the compression $\gamma$ and sample a new hypergraph $\textsf{H}'$ from the distribution $p(\cdot ~|~\gamma )$. We can think of this process as describing the hypergraph $\textsf{H}$ by transmitting the compression $\gamma$ to a stranger who does not observe $\textsf{H}$ itself. The stranger then forms a guess $\textsf{H}'$ about the structure of the hypergraph described by the compression.

We seek a distribution *q* over $\Gamma$ that maximizes the mutual information between $\textsf{H}$ and $\textsf{H}'$:

1 $$\begin{aligned} q = {\mathop {\mathrm{arg\,max}}\limits _q}\, I(\textsf{H}; \textsf{H}') \quad \text {such that} \quad \textsf{H}' \sim p(\cdot ~|~\gamma ) \quad \text {and} \quad \gamma \sim q\;. \end{aligned}$$

To simplify this problem, we first observe that, by construction, $\textsf{H}$ and $\textsf{H}'$ are independent conditioned on $\gamma$:

$$\begin{aligned} p(\textsf{H}, \textsf{H}'~|~\gamma ) = p(\textsf{H}'~|~\textsf{H}, \gamma )p(\textsf{H}~|~\gamma ) = p(\textsf{H}'~|~ \gamma )p(\textsf{H}~|~\gamma )\;. \end{aligned}$$

The last equality reflects the fact that, once $\gamma$ is transmitted, the signal receiver does not have any other access to $\textsf{H}$ when generating the guess $\textsf{H}'$. Now applying the chain rule of mutual information, we have

$$\begin{aligned} I(\textsf{H};\textsf{H}') = I(\textsf{H}; \gamma , \textsf{H}') - I(\textsf{H}; \textsf{H}'|\gamma )\;. \end{aligned}$$

By conditional independence, $I(\textsf{H}; \textsf{H}'|\gamma ) = 0$ and $I(\textsf{H}; \gamma , \textsf{H}') = I(\textsf{H}; \gamma )$. It follows that

$$\begin{aligned} I(\textsf{H}; \textsf{H}') = I(\textsf{H}; \gamma ) = H(\textsf{H}) - H(\textsf{H}~|~\gamma )\;. \end{aligned}$$

Since the first term does not depend on $\gamma$, we can ignore it in the optimization over *q*, and our reduced problem becomes

$$\begin{aligned} q = {\mathop {\mathrm{arg\,min}}\limits _q}\, H(\textsf{H}~|~\gamma ) \quad \text {such that} \quad \gamma \sim q\;. \end{aligned}$$

Expanding the conditional entropy yields

$$\begin{aligned} H(\textsf{H}~|~ \gamma )&= \sum _{\gamma \in \Gamma }\sum _{\textsf{H}\in \mathbb {H}(\gamma )} p(\textsf{H}, \gamma ) \log p(\textsf{H}~|~\gamma ) \\&= \sum _{\gamma \in \Gamma } q(\gamma ) \sum _{\textsf{H}\in \mathbb {H}(\gamma )} p(\textsf{H}~|~ \gamma ) \log p(\textsf{H}~|~\gamma )\;. \end{aligned}$$

This expression makes clear that the optimal *q* concentrates all its mass on values $\gamma$ that minimize the entropy of the distribution $p(\cdot ~|~ \gamma )$. But since $p(\cdot ~|~ \gamma )$ is uniform, the entropy of this distribution is simply $\log Z(\gamma )$, where $Z(\gamma ) = \left| \mathbb {H}(\gamma )\right|$ is the number of hypergraphs compatible with $\gamma$. Thus, after observing a data hypergraph $\textsf{H}_0$ and setting $\Gamma = \Gamma (\textsf{H})$, our original mutual information maximization problem Eq. (1) reduces to the problem

2 $$\begin{aligned} \hat{\gamma } = {\mathop {\mathrm{arg\,min}}\limits _{\gamma \in \Gamma (\textsf{H}_0)}}\, Z(\gamma )\;. \end{aligned}$$

That is, the maximally informative compression $\gamma$ of a given hypergraph $\textsf{H}_0$ is the compression that is compatible with $\textsf{H}_0$ and minimizes the size of $\mathbb {H}(\gamma )$. We can think of $\gamma$ as a description of $\textsf{H}_0$ that minimizes the number of alternative hypergraphs $Z(\gamma )$ which could also be described by $\gamma$.

### Relation to maximum-likelihood estimation

The entropy minimization problem of Eq. (2) and maximum-likelihood estimation arise from the stochastic blockmodel. Recall the conditional data generating distribution $p(\cdot ~|~\gamma )$, which is uniform over the set $\mathbb {H}(\gamma )$ of all hypergraphs compatible with the compression $\gamma$:

$$\begin{aligned} p(\textsf{H}~|~\gamma ) = {\left\{ \begin{array}{ll} \frac{1}{Z(\gamma )} \quad \textsf{H}\in \mathbb {H}(\gamma ) \\ 0 \quad \text {otherwise.} \end{array}\right. } \end{aligned}$$

We can then equivalently write our minimum-entropy problem as:

3 $$\begin{aligned} \gamma = {\mathop {\mathrm{arg\,min}}\limits _{\gamma \in \Gamma (\textsf{H}_0)}}\, Z(\gamma ) = {\mathop {\mathrm{arg\,max}}\limits _{\gamma \in \Gamma (\textsf{H}_0)}}\, \frac{1}{Z(\gamma )} = {\mathop {\mathrm{arg\,max}}\limits _{\gamma \in \Gamma (\textsf{H}_0)}}\, p(\textsf{H}~|~\gamma )\;. \end{aligned}$$

Since $\gamma$ itself contains cluster memberships and edge-cluster intersections, $p(\cdot ~|~\gamma )$ can be viewed as a microcanonical hypergraph stochastic blockmodel, generalizing known microcanonical models for graphs^11^. The mutual information maximization Eq. (1), the entropy minimization Eq. (2), and the maximum-likelihood problem Eq. (3) are all equivalent ways to describe our inference problem.

One can count the number of graphs *G* that admit $\gamma = (\left\{ C_i\right\} _{i=1}^m, \mathbb M)$ as a compression, where $\left\{ C_i\right\} _{i=1}^m$ is a partition of the vertex set of *G* and each entry of the module matrix $\mathbb M_{i,j}$ enumerates the number of edges between cluster *i* and *j*, as follows:

$$\begin{aligned} Z(\gamma ) = \prod _{i<j} \left( {\begin{array}{c}\left| C_i\right| \left| C_j\right| \\ \mathbb M_{i,j}\end{array}}\right) \prod _{i=1}^m \left( {\begin{array}{c}\left( {\begin{array}{c}\left| C_i\right| \\ 2\end{array}}\right) \\ \mathbb M_{i,i}\end{array}}\right) . \end{aligned}$$

Our aim is to maximize the mutual information between a hypergraph $\textsf{H}$ and its compression. To do this via Eq. (2), we need to evaluate $Z(\gamma )$, the number of hypergraphs compatible with the compression $\gamma$. If we restrict to *simple* hypergraphs, which do not have multiple edges, then

$$\begin{aligned} Z(\gamma ) = \prod _{\lambda \in \mathbb N^m}\left( {\begin{array}{c}\prod _{i=1}^m \left( {\begin{array}{c}\left| C_i\right| \\ \lambda _i\end{array}}\right) \\ e_\lambda \end{array}}\right) . \end{aligned}$$

We remark that the (a priori) infinite limit exists, as all but finitely many $\lambda$ are $\textbf{0}$. Here, the expression $$\prod _{i=1}^m \left( {\begin{array}{c}\left| C_i\right| \\ \lambda _i\end{array}}\right)$$ counts the number of ways to choose the appropriate number of vertices from each of the *m* clusters for inclusion in one $\lambda$-edge, from which we select $e_\lambda$ edges without repetition to realize.

If we instead consider multi-hypergraphs, in which multiple edges are permitted, then there are

$$\left( \prod _{i=1}^m \left( {\begin{array}{c}\left| C_i\right| \\ \lambda _i\end{array}}\right) \right) ^{e_\lambda } =\prod _{A \in E_\lambda }\prod _{i=1}^m \left( {\begin{array}{c}\left| C_i\right| \\ \lambda _i\end{array}}\right)$$

ways to select the $e_\lambda$ edges from among all possible edges of type $\lambda$. It follows in this case that

$$\begin{aligned} Z(\gamma )= \prod _{\lambda \in \mathbb N^m} \prod _{A\in E_\lambda } \prod _{i=1}^m \left( {\begin{array}{c}\left| C_i\right| \\ \lambda _i\end{array}}\right) . \end{aligned}$$

Noting that $\lambda _i=\left| A \cap C_i\right|$ if $A \in E_\lambda$, we can rewrite this expression as

$$\begin{aligned} Z(\gamma ) = \prod _{\lambda \in \mathbb N^m} \prod _{A\in E_\lambda } \prod _{i=1}^m \left( {\begin{array}{c}\left| C_i\right| \\ \left| A\cap C_i\right| \end{array}}\right) = \prod _{A\in E} \prod _{i=1}^m \left( {\begin{array}{c}\left| C_i\right| \\ \left| A\cap C_i\right| \end{array}}\right) . \end{aligned}$$

Notably, this final expression is not organized according to edge type.

### Degree-corrected entropy

In this section we vary the compression to allow for specification of a degree sequence in the hypergraph. In doing so, we will obtain a new entropy based objective function to minimize. As in the previous section, this entropy will be inspired by a hypergraph counting task.

We consider degree-corrected compressions of the form $\gamma = (\left\{ C_i\right\} _{i=1}^m,\left\{ e_\lambda \right\} _{\lambda \in \mathbb N^{m}},\left\{ d_i\right\} _{i\in V})$. We again let $Z(\gamma )$ denote the number of hypergraphs compatible with $\gamma$ as a degree-corrected compression. We again seek to maximize mutual information by minimizing $Z(\gamma )$, which again requires a formula for $Z(\gamma )$.

Let

$$\begin{aligned} e_i = \sum _{\lambda \in \mathbb N^m} \lambda _ie_\lambda \end{aligned}$$

for $1\le i \le m$ denote the degree sum of vertices in cluster $C_i$. In what follows, we treat degrees as distinguishable “stubs” hanging off of vertices. We imagine constructing a hypergraph $\textsf{H}$ with the desired compression $\gamma$ through the following process:

1. First, assign the available stubs within each cluster $C_i$ to the $\lambda$-types to which they will contribute.
2. Second, for each $\lambda$-type:
  - for each $1\le i \le m$, group the assigned stubs from cluster $C_i$ into packets of size $\lambda _i$, then
  - combine the packets into edges of $\lambda$-type.

To count the number of hypergraphs compatible with $\gamma$, it suffices to count the number of possible $\lambda$-type assignments, $a(\gamma )$, from which to choose in Step 1, and then for each $\lambda \in \mathbb N^m$, the number of possible packets, $p_\lambda (\gamma )$, from which to choose in Step 2(a) and the number possible combinations of these packets into edges, $c(\gamma )$, in Step 2(b).

The first assignment step can be done in

4 $$\begin{aligned} a(\gamma )= \prod _{i=1}^m \left( {\begin{array}{c}e_i\\ \ldots ,\lambda _ie_\lambda ,\ldots \end{array}}\right) \end{aligned}$$

possible ways, where the lower portion of the multinomial coefficient ranges over all $\lambda \in \mathbb N^m$.

To proceed with the second step, suppose $\lambda$ is fixed. Notice that each edge of $\lambda$-type requires $\lambda _i$ degrees from cluster *i*. Furthermore, recall that in the first step, we allocated $\lambda _ie_\lambda$ degrees for the purpose of construction $\lambda$-type edges. We can group the $\lambda _ie_\lambda$ degrees into packets of size $\lambda _i$ in $$\left( {\begin{array}{c}\lambda _ie_\lambda \\ \ldots , \lambda _i,\ldots \end{array}}\right)$$ ways, where the lower portion of the multinomial coefficient is repeated $e_\lambda$ times. Note that the packets produced by multinomial coefficients are ordered, which we will account for later. Repeating this process for each cluster completes Step 2(a) and can be done in a total of

5 $$\begin{aligned} p_\lambda (\gamma )=\prod _{i=1}^m \left( {\begin{array}{c}\lambda _ie_\lambda \\ \ldots , \lambda _i,\ldots \end{array}}\right) \end{aligned}$$

ways.

There is a natural way to combine packets into edges: simply take the first packet from each cluster to produce the first edge, then take the second packet from each cluster to produce the second edge, and so on. (Note that if $\lambda _i=0$, we proceed as if there is an infinite stream of empty packets.) Notice that the same set of edges can be produced in $e_\lambda !$ ways. We account for this by dividing our count by $e_\lambda !$, which resolves the fact that the multinomial coefficients counted ordered packets. This essentially finishes step 2(b), which when combined with the expression from (5) for each $\lambda$ produces

6 $$\begin{aligned} c(\gamma )= \prod _{\lambda \in \mathbb N^m}\left( (e_\lambda !)^{-1}(p_\lambda (\gamma )\right) = \prod _{\lambda \in \mathbb N^m}\left( (e_\lambda !)^{-1}\prod _{i=1}^m \left( {\begin{array}{c}\lambda _ie_\lambda \\ \ldots , \lambda _i,\ldots \end{array}}\right) \right) . \end{aligned}$$

Therefore, combining expression (4) and (6) and forgetting the degree stub labels gives

7 $$\begin{aligned} Z(\gamma )&= a(\gamma ) c(\gamma )\nonumber \\&= \prod _{i=1}^m \left( {\begin{array}{c}e_i\\ \dots ,\lambda _ie_\lambda ,\dots \end{array}}\right) \prod _{\lambda \in \mathbb N^m}\left( (e_\lambda !)^{-1}\prod _{i=1}^m \left( {\begin{array}{c}\lambda _ie_\lambda \\ \dots , \lambda _i,\dots \end{array}}\right) \right) \nonumber \\&=\frac{\prod _{i=1}^m e_i!}{\left( \prod _\lambda e_\lambda !\right) \left( \prod _{\lambda }\prod _{i=1}^m (\lambda _i!)^{e_\lambda }\right) }. \end{aligned}$$

**Figure 2:**
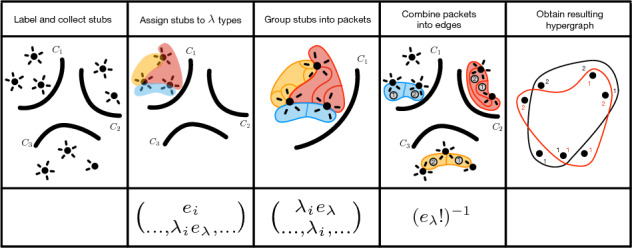
Visualization of each of the constituent counting steps for Eq. (7). Here, bold curves demarcate clusters, colored regions illustrate assigning stubs to $\lambda$ types, and colored curves illustrate packet assignment.

An important remark is that we have technically counted hypergraphs $\textsf{H}$ where we allow vertices to appear multiple times in an edge. This is a choice we make to simplify the hypergraph counts. By distinguishing the stubs attached to each vertex from each other, we have also overcounted hypergraphs with parallel hyperedges. Equation (7) gives therefore an *approximation* of the exact degree-corrected entropy. The quality of this approximation depends on the statistical prevalence of multiple vertex inclusions and parallel hyperedges^16^. In graphs with fixed degree sequences, it is known that, provided that the low-order moments of the degree sequence remain constant as the number of vertices grows large (i.e. in the “large, sparse limit”), the number of multiple inclusions and parallel edges is concentrated around constants that depend on moments of the degree sequence^17^. It follows that the proportion of edges with multiple vertex inclusions or with parallel edges approaches zero in the limit. We are unaware of formal proofs of similar results for hypergraphs or for graphs with community structure. We conjecture that the same heuristic should roughly hold: provided that the degree sequence and edge-size sequence of the hypergraph have low-order moments that are sufficiently small relative to the number of vertices, the approximate entropy will be very close to the exact entropy. A visualization of each of the constituent steps for producing Eq. (7) can be found in Fig. 2.

In light of allowing vertices to appear multiple times in an edge and the form of Eq. (7), it is tempting to assume that the degree sequence of $\textsf{H}$ does not impact the entropy calculation. This is partially correct. The degrees matter up to cluster assignment; which is to say that the entropy calculation varies with the total degrees of the clusters, but not with the degree distribution within the clusters. However, the particular degree sequence of $\textsf{H}$ does influence how the entropy calculation acts across the whole state space. The entropy of two cluster assignments which differ by a single vertex *v* is a function of the degree of *v* and the cluster placement of the neighbors of *v*. In other words, the degree of *v* determines how the total degrees of clusters change when we change the cluster assignment of *v*. This, in turn, determines how entropy changes.

### Simulated annealing

Our aim is to cluster a hypergraph $\textsf{H}$ by selecting the partition $\left\{ C_i\right\} _{i=1}^m$ which maximizes the mutual information between $\textsf{H}$ and the compression $\gamma$ induced by $\left\{ C_i\right\} _{i=1}^m$. For this section, it is convenient to instead use the vector representation ${\textbf {c}}\in \mathbb {Z}^n$, where $c_j$ gives the cluster to which vertex *j* is assigned by the partition $\left\{ C_i\right\} _{i=1}^m$. A choice of ${\textbf {c}}$ is equivalent to a choice of partition $\left\{ C_i\right\} _{i=1}^m$ and therefore to a choice of compression $\gamma$. Hence, we can define the entropy $H({\textbf {c}})$ and number of compatible hypergraphs $Z({\textbf {c}})$. We aim to minimize $Z({\textbf {c}})$. Performing this minimization exactly is computationally intractable, even for dyadic networks^10^. We therefore perform approximate stochastic optimization via simulated annealing^18^.

To perform simulated annealing, we use the Metropolis-Hastings algorithm^19^ to construct a random walk on the space of candidate clusterings. We begin at a uniformly random clustering ${\textbf {c}}^{(0)} \in \mathbb {Z}^n$. At each timestep *t*, we select a vertex and candidate label $(v,i) \in V\times \left\{ 1,\dots ,m\right\}$ uniformly at random and propose a new state state ${\textbf {c}}'$ where $c_u'=c_u^{(t)}$ for $u \ne v$ and $c_v'=i$. Let $\Delta ({\textbf {c}}', {\textbf {c}}) = \log Z({\textbf {c}}') - \log Z({\textbf {c}})$. We accept ${\textbf {c}}'$ as the new state with probability $\min \left\{ 1,e^{-\beta \Delta ({\textbf {c}}', {\textbf {c}})}\right\}$ and reject ${\textbf {c}}'$ otherwise, where $\beta \ge 0$ is an *inverse temperature* parameter. If ${\textbf {c}}'$ is accepted, then we set ${\textbf {c}}^{(t+1)} = {\textbf {c}}'$. From standard results on the Metropolis-Hastings algorithm, this random walk has a stationary distribution and the mass of this distribution at ${\textbf {c}}$ is proportional to $Z({\textbf {c}})^{-\beta }$. The mode(s) of this distribution occur at the value(s) of ${\textbf {c}}$ that minimize $Z({\textbf {c}})$, with the sharpness of these modes depending on the inverse temperature $\beta$. For small $\beta$, much of the probability mass of the stationary distribution lies away from the modes, whereas as $\beta \rightarrow \infty$ the mass concentrates on these modes. In simulated annealing, we allow $\beta = \beta (t)$ to depend on the timestep, gradually increasing $\beta (t)$ as the algorithm proceeds.

We use $\beta (t)=(t+1)\cdot 0.0001$ for all of our applications, as this seems to work better than $(t+1)\cdot 0.001$ or $(t+1)\cdot 0.00001$. An important note is that we allow the proposed cluster assignment of a vertex to be the same cluster assignment it already has (that is ${\textbf {c}}'={\textbf {c}}$ is allowed in our implementations). Therefore, if *m* is the number of clusters, then about 1/*m* of all steps our algorithm proposes do not change the clustering.

Because we aim to find minima rather than sample from the stationary distribution, we track the cluster assignment vector that minimizes entropy along our random walk. For pseudocode, see Algorithm 1.

**Algorithm 1:**
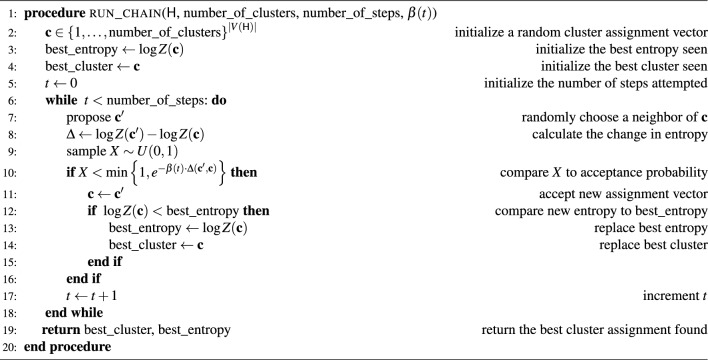
This algorithm will use simulated annealing to find a cluster assignment with low entropy. Note that *Z* implicitly depends on the hypergraph $\textsf{H}$.

### Model selection

The proposed clustering procedure here requires a given number of clusters. Although there may be *a priori* well-reasoned choices for sensible values of *m*, the number of clusters to cluster a given hypergraph into, there is no guarantee the interested practitioner will have a selected *m* in mind. Should *m* be difficult to choose or unknown *a priori*, we then find ourselves faced with a model selection problem before we may even begin clustering.

While a variety of approaches have been proposed for choosing the optimal number of communities into which to cluster a (hyper)graph^20^, our method suggests an information-theoretic approach: utilizing the principle of parsimony and choosing an appropriate number of clusters *m* given the clustering’s description length^10,21,22^. In this framework, a principled choice for *m*, unless otherwise constrained by domain knowledge or hypothesis, is the value that minimizes total description length. If we express by $L(\textsf{H})$ the total number of bits to precisely describe $\textsf{H}$, then we can decompose $L(\textsf{H})$ as

8 $$\begin{aligned} L(\textsf{H}) = L(\gamma ) + L(\textsf{H}\mid \gamma ) \end{aligned}$$

where $\textsf{H}$ is a given hypergraph and $\gamma$ is a proposed compression of $\textsf{H}$. Hence, our model selection can be performed via the entropy-parsimonious minimum description length value for *m* given as the solution to the equation

9 $$\begin{aligned} m^* = \underset{m}{\text {argmin}} \, \Big [ L(\gamma ) + L(\textsf{H}\mid \gamma _m) \Big ] \end{aligned}$$

where $\gamma _m$ is the optimal compression of $\textsf{H}$ into *m*-many clusters with our proposed method.

We expand Eq. (8) as

10 $$\begin{aligned} L(\gamma ) + L(\textsf{H}\mid \gamma ) = n\log m + \sum \limits _{k=2}^{k^*} \left( {\begin{array}{c}m + k - 1\\ k\end{array}}\right) \log \ell _k + H(\textsf{H}\mid \gamma ) \end{aligned}$$

where *n* is the number of vertices in the hypergraph, *m* is the number of groups in partition $\gamma$, $\ell _k$ is the number of hyperedges of size *k*, and $k^*$ is the size of the largest hyperedge in $\textsf{H}$.

The description length under this coding scheme is known to frequently underestimate the number of clusters as compared to ground truth from a given generative model^10^. Our work is consistent with these findings. However, the minimum description length provides some amount of insight and acts as a counterweight to uninformed selection of the number of clusters. We report the results applied to the real systems in Table 1.

**Table 1:** Selecting the number of clusters via the minimum description length principle.

Data set	*m*	Average	SD	Minimum
High school	4	126678	955.498	124704
	5	125516	1030.96	123736
	**6**	**124840**	**1074.93**	**123442**
	7	125150	978.124	123946
	8	126557	590.183	125368
	9	129394	583.888	128302
Primary school	4	239454	1122.45	237264
	5	237048	805.445	235960
	**6**	**236618**	**726.493**	**235495**
	7	236923	497.284	236165
	8	238765	679.516	237889
	9	241589	725.895	240501
	10	245773	501.992	245034
	11	251477	446.934	250660
	12	259522	498.737	258784
MTG	2	4.440 $\times 10^7$	402.753	4.440 $\times 10^7$
	**3**	**4.435 ** $\times \varvec{10^7}$	**156455**	**4.413 ** $\times \varvec{10^7}$
	4	4.514 $\times 10^7$	21227.6	4.512 $\times 10^7$
	5	$\underline{5.690 \times 10^7}$	$\underline{1.501 \times 10^{-5}}$	$\underline{5.690 \times 10^7}$
	6	1.511 $\times 10^8$	1.128 $\times 10^{-5}$	1.511 $\times 10^8$

The average, standard deviation, and minimum calculated description lengths (in bits) of 10 independent clusterings are presented for the real data sets. The suggested number of clusterings—on the basis of average description length—is bolded for each data set while the ground truth is underlined. Note that the MTG data set “ground truth” is discussed further in Section “Clustering magic: the gathering cards”.

## Results: synthetic data

The stochastic blockmodel is a method to generate random graphs with latent community structure. For a review of the stochastic blockmodel in simple graphs, see the work by Lee and Wilkonson^23^. Given vertex sets $V_1, \ldots , V_m$ with sizes $n_1,\ldots , n_m$ respectively, we want to generate a hypergraph on the vertex set $\bigcup _{1\le i \le m} V_i$, where each $V_i$ is a latent community within the graph. In order to do this, we add a hyperedge of $\lambda$-type with probability $P_\lambda$. Communities may be denser or sparser depending on the choice of the probabilities $P_\lambda$.

We generate hypergraphs according to the following parameters: two ground truth communities of size $n=200$, where each vertex sees on average five 2-edges and five 3-edges. This means we must generate exactly 5*n* 2-edges and $\tfrac{10}{3}n$ 3-edges. We generate these edges so that the total proportion of 2-edges within one of the two latent clusters is $p_2$ and the total proportion of 3-edges within one of the two clusters is $p_3$, for various choices of $0\le p_2,p_3\le 1$. This model roughly corresponds to choosing $P_{(0,2)}=P_{(2,0)}$ with $P_{(0,2)}+P_{(1,1)}=\tfrac{5}{2n}$, and $P_{(3,0)}=P_{(0,3)}$, $P_{(2,1)}=P_{(1,2)}$ with $P_{(3,0)}+P_{(1,2)} = \tfrac{10}{n^2}$ where a bit more care needs to be taken to balance the number of edges within communities and between communities. The advantage of not strictly following the stochastic blockmodel is that synthetic hypergraphs can be generated in $O(n\log n)$ steps (in the number of vertices) time rather than cubic time.

The parameters we use for our synthetic data are the same parameters used in previous literature^5,8^. The number of vertices is chosen for pragmatic reasons; larger graphs take longer to process. The average degrees are fixed to 5 in our paper to have directly comparable results with the non-backtracking spectral method and the belief-propagation method^8^. The more important parameters are the relative distribution of edges within and between clusters, governed by $p_2$ and $p_3$, for which we do an exhaustive sweep.

**Figure 3:**
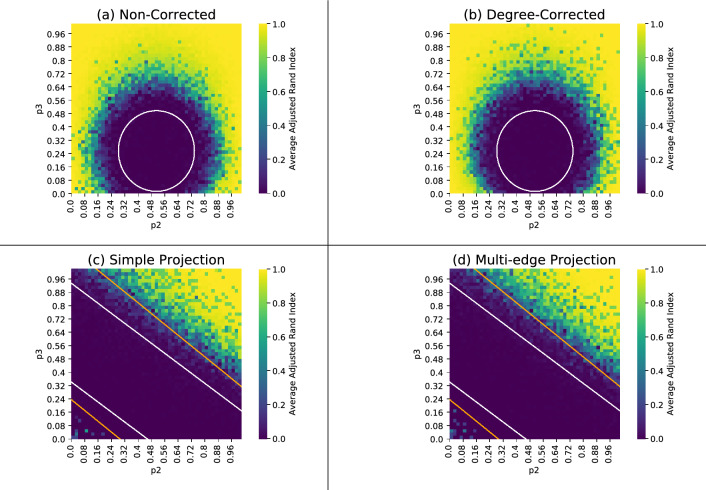
Each heatmap has $51\times 51$ pixels, where each pixel represents the average ARI across 5 hypergraphs. Each hypergraph underwent 20 independent clustering attempts, of which we used the results from the run which achieved the lowest entropy. The same hypergraphs are used across all four plots. The white ellipse in plots (**a**) and (**b**) are the conjectured detection threshold for Belief-Propagation Spectral Clustering for hypergraphs^8^. The white lines in plots (**c**) and (**d**) are the conjectured detection threshold for Non-Backtracking Spectral Clustering for hypergraphs^8^. The orange lines in plots (**c**) and (**d**) are the proven detection threshold for the graph stochastic blockmodel^28^ for edge densities of the multi-edge projection parameterized by $p_2$ and $p_3$.

The heatmaps in Fig. 3 show the results of a series of experiments on the planted partition model described above. Each pixel gives the average ARI (Adjusted Rand Index) of the cluster assignments found by our algorithm compared to the planted partition after 20 attempts, for varying parameters of $p_2, p_3$. In these visualizations, the region bounded by the white curves is the detectability threshold for hypergraph spectral methods conjectured by Chodrow, Eikmeier, and Haddock^8^. In other contexts, the detectability threshold phenomenon is phrased as estimator consistency^24–27^. While our present results fall short of these conjectured thresholds, we note that these thresholds were derived under the Nishimori Condition, which assumes that the edge probabilities and the number of latent communities in the stochastic blockmodel are known exactly. In contrast, our proposed method does not require estimation or knowledge of the edge probabilities, though we do assume that the number of clusters are known.

**Figure 4:**
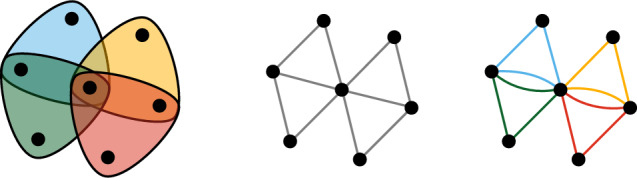
From left to right, a hypergraph, its simple clique projection, and its multi-edge clique projection.

We compare the performance of our algorithm on these planted-partition hypergraphs to its performance on the simple and multi-edge projections. The simple projection of a hypergraph is a dyadic graph on the same vertex set, wherein a simple pairwise edge connects each pair of vertices that participate together in some hyperedge. This projection is a lossy representation of a hypergraph since two vertices are connected by at most one dyadic edge, whether they participate in one hyperedge together or many. For this reason, we also consider the multi-edge projection, wherein a pair of vertices that participate in *k* distinct hyperedges are connected by *k* dyadic edges in the expansion (or equivalently, a single dyadic edge with edge weight *k*). See Fig. 4 for an example.

**Figure 5:**
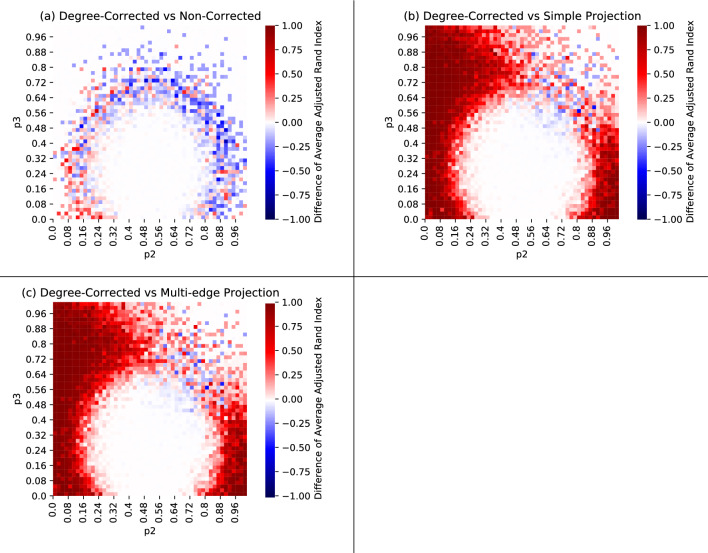
Plot (**a**) compares the degree-corrected hypergraph chain against the non-corrected chain. Interestingly, $p_2,p_3\in [0.00,0.32]$ range appears to favor the degree-corrected chain, while the rest of the circular boundary region favors the non-corrected chain. Plots (**b**) and (**c**) are a comparison of hypergraph native results with multi-edge and simple projection results; red indicates a superior performance by the degree-corrected hypergraph chain, while blue indicates that the degree-corrected algorithm on the projection performed better.

Plots (c) and (d) in Fig. 3 shows the results of our degree-corrected algorithm using the simple and multi-edge projections, respectively. The orange lines are the detection thresholds for the graph stochastic blockmodel^28^ using the edge densities of the multi-edge projection parameterised by $p_2$ and $p_3$. Since the hypergraphs we generated are sparse, there should be relatively few multi-edges in the multi-edge projection, suggesting that the edge densities in the multi-edge and simple projections are similar. This also justifies using the detection threshold for the graph stochastic blockmodel, which holds for sparse hypergraphs. Interestingly, both the simple and multi-edge projection find some success within the detection threshold, suggesting that some mutual information clustering may be sensitive to some of the latent hypergraph information in the projections. For example, the presence of triangles in the projections of a sparse hypergraph are potentially distinguishing from the graph stochastic blockmodel or the sparse Erdős-Rényi random graph. A comparison between the degree-corrected algorithm’s performance on multi-edge and simple projections can be found in Fig. 5.

### Results: degree correction on synthetic data

In order to determine whether the degree-corrected chain performs better than a non-corrected chain, we generated a synthetic hypergraph with heterogeneous vertex degrees that are not informative of the ground truth clustering. We roughly follow the degree-corrected hypergraph stochastic blockmodel (DCHSBM) as presented by Chodrow, Veldt, and Benson^5^, with a few modifications to make the generated graph amenable to the non-corrected entropy calculations used in the non-corrected chain.

We generated our hypergraph as follows. Our hypergraph has 2 ground truth clusters with 50 vertices in each cluster. For each sub-multiset *R* of size 2 or 3 of the vertices (allowing for multiple vertices in an edge), we sample

$$\begin{aligned} X_R\sim {\textbf {Possion}}\left( \prod _{v\in R}\theta _v\cdot \Omega (R)\right) \end{aligned}$$

where $\theta _v$ is a parameter controlling the expected degree in the DCHSBM, and $\Omega$ is an intensity function akin to $P_\lambda$ in our hypergraph stochastic blockmodel. We use $\theta _v = 1/r_v$ where $r_v$ is a uniformly chosen random integer between 1 and 24. We use the all-or-nothing intensity function given by

$$\begin{aligned} \Omega (R) = {\left\{ \begin{array}{ll}1&{}\text { if all { R} is contained a single ground truth cluster}\\ 0.1&{} \text {otherwise}\end{array}\right. }. \end{aligned}$$

If $X_R>0$, then we include *R* as an edge in our sampled hypergraph; this is the first difference between our generation method and the DCHSBM as presented in Chordow, Veldt, and Benson^5^, since the DCHSBM includes the edge *R* with multiplicity $X_R$. Unfortunately, multi-edge pose an issue for the non-corrected entropy calculation, making this change necessary for our experiment. Finally, there is a possibility that an edge *R* contains only a single vertex with some multiplicity. These edges also pose a problem for the non-corrected entropy calculation. Therefore, to make our hypergraph amenable to the non-corrected entropy calculation, we replace every edge with a single vertex *v* (with some multiplicity) by an edge of size 2 containing the vertices *v* and $(v+1) \mod 100$. The hypergraph that we sample and use for our experiment has 100 vertices evenly split between 2 ground truth clusters with 450 2-edges and 1296 3-edges.

**Figure 6:**
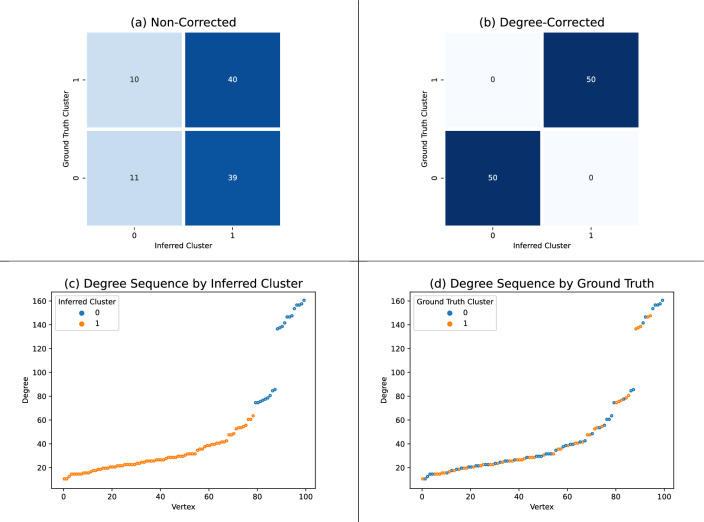
The non-corrected and degree-corrected chains were given 10 runs with 20, 000 steps, where we keep the inferred clustering with maximum mutual information observed over all runs. Plot (**a**) shows the inferred clusters from the non-corrected chain, which scored an ARI of $-0.006$. Plot (**b**) shows the inferred clusters from the degree-corrected chain, which scored an ARI of 1.00. Plot (**c**) shows the degrees of vertices (vertices are sorted by degree), with a color-coding that corresponds to the inferred cluster from plot (**a**). Plot (**d**) shows the degrees of vertices with a color-coding that corresponds to the ground truth cluster.

Figure 6 presents the inferred clusters on our sampled hypergraph using the non-corrected and degree-corrected chains. The non-corrected chain does not recover the ground truth clusters and scored an ARI of $-0.006$. The degree-corrected chain recovered the ground truth clusters perfectly with an ARI of 1.00. In order to test the hypothesis that the non-corrected chain fails to recover the ground truth clusters because of the uninformative heterogeneous vertex degrees, we compare the degrees of vertices in the inferred clusters. Figure 6c shows the degrees of vertices (sorted by degree) with a color-coding that corresponds to the clusters inferred by the non-corrected chain. This plot does not rule out the hypothesis that the non-corrected chain infers clusters that co-vary with the degrees of vertices. For comparison, Fig. 6d shows the degrees of vertices (sorted by degree) with a color-coding that corresponds to the ground truth clusters; this plot shows that both of the ground truth clusters have a similar degree distribution that contains both high and low degree vertices. As a result, we find that this synthetic experiment supports our claim that the degree-correct chain can succeed where the non-corrected chain fails, because of the degree-correction.

### Results: comparison to spectral clustering

**Figure 7:**
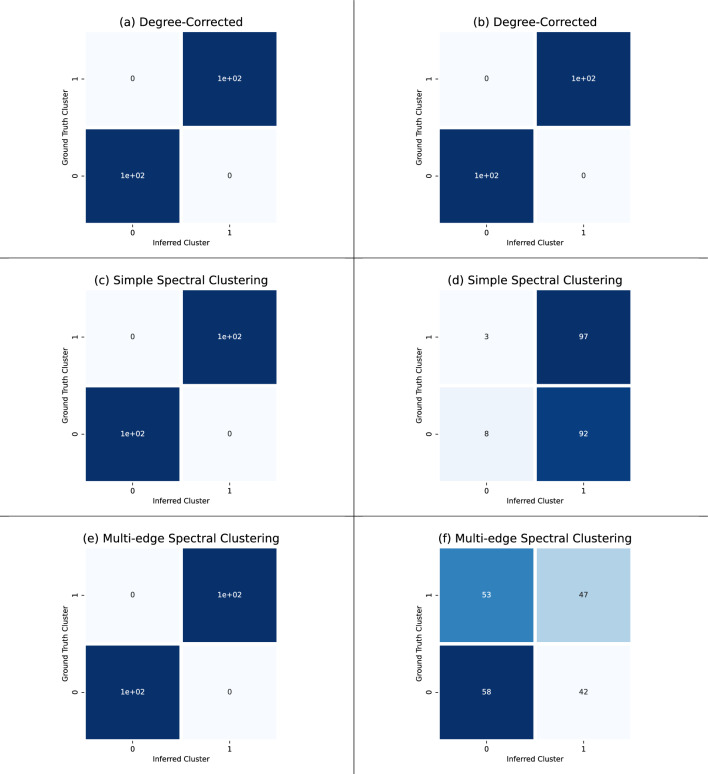
On the left hand side, we applied the degree-corrected mutual information clustering (best of 20 runs with 20, 000 steps), simple projection spectral clustering, and multi-edge projection spectral clustering on a 200 vertex hypergraph stochastic block model sampled with parameters $p_2 = 1$ and $p_3 = 0.97$; this achieved ARIs of 1.00, 1.00, and 1.00 for (**a**), (**c**), and (**e**), respectively. On the right hand side, we applied the degree-corrected mutual information clustering, simple projection spectral clustering, and multi-edge projection spectral clustering on a 200 vertex hypergraph stochastic block model sampled with parameters $p_2 = 1$ and $p_3 = 0$; this achieved ARIs of 1.00, 0.00, and 0.00 for (**b**), (**d**), and (**f**), respectively. Representative run times for the degree-corrected, simple projection spectral clustering, and multi-edge projection spectral clustering in seconds are 35.551 s, 0.036 s, and 0.043 s, respectively.

We compared the degree-corrected mutual information clustering to spectral clustering on the simple and multi-edge clique projection for two different parameter settings in our hypergraph stochastic block model. The first parameter setting we used is $p_2 =1$ and $p_3 = 0.97$. Figure 5 predicts that both hypergraph and graph with projections should solve this clustering task. Figure 7a,c,e confirm this.

On the other hand, sampling from the stochastic block model with parameters $p_2=1$, $p_3=0.97$ should result in a hypergraph whose clusters information is lost after either a simple or multi-edge projection (see Fig. 5). Figure 7b,d,f shows that the degree-corrected mutual information clustering method can recover the clusters while spectral clustering applied to the simple and multi-edge projection cannot.

Using the hypergraph information comes at the cost of time where the degree-corrected mutual information clustering required 35.551 seconds while the spectral methods required less than 0.05 seconds. Given that our hypergraphs are relatively small (with only 200 vertices), run time is a concern for mutual information clustering. However, this experiment provides further evidence that there are hypergraph clustering problem instances that cannot be solved by simple graph or multi-graph methods.

## Results: experiments on data

### Primary school contact hypergraph

**Figure 8:**
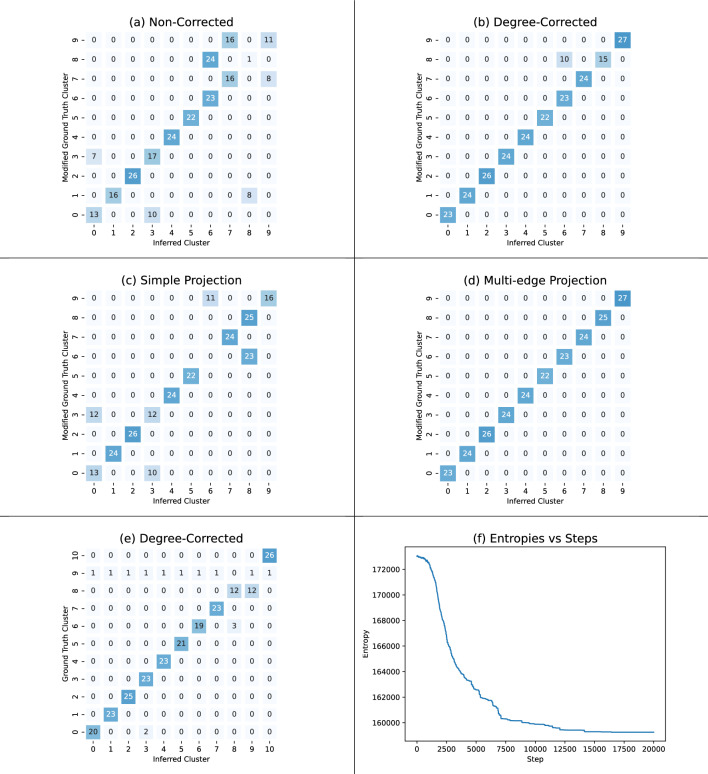
Inferred and ground-truth clusters for the primary school contact data set^29^. Each matrix is the lowest entropy of 50 independent runs of 20,000 steps. The cluster heatmap for (**e**) uses collected ground truth; this includes a cluster label for teachers within the primary school. The cluster heatmaps for (**b**), (**c**), and (**d**) are compared against a modified ground truth where the teachers’ cluster from the ground truth is divided up among the classrooms according to the assignments in (**a**). The ARI values are (**a**) 0.66, (**b**) 0.93, (**c**) 0.76, (**d**) 1.00, and (**e**) 0.88. The line graph in (**f**) shows the entropy at each step of the simulated annealing minimization process. The sharp drop in entropy is typical behavior. We tuned the number of steps and the annealing schedule so that the early portion of simulation explores the state space, the descent is (relatively) gradual, and the end of the simulation settles into a local minimum.

The primary school contact data set obtained from Stehlé et al.^29^ provides a hypergraph with 242 vertices. Edges in this hypergraph correspond to groups of students and teachers that were within 1.5 m of each other and facing each other. The ground truth for this data set assigns students to one of 10 classrooms, while teachers are all assigned to their own cluster. Running our algorithm on this data using 11 clusters resulted in an ARI of 0.88 after selecting the lowest-entropy cluster assignment from 50 runs with 20, 000 steps each (Fig. 8e). We also studied our model’s performance on a modified version of the data set in which each teacher vertex is given the label of their classroom, resulting in 10 clusters. The non-degree corrected algorithm did not perform well on this modified data set (scoring an ARI of 0.66 in Fig. 8a). Running our degree corrected algorithm on the modified data set cluster recovery with an ARI of 0.93, again after 50 runs with 20, 000 steps (Fig. 8b).

We compared our algorithm to two simulated annealing algorithms defined on projections of the data. A chain on a simple graph projection obtained an ARI of 0.76 (Fig. 8c), while a chain defined on a multi-edge projection scored an ARI of 1.00 (Fig. 8d). These results indicate the value of higher-order relationships in clustering hypergraph data and are qualitatively aligned with prior hypergraph algorithms applied to this data set^5^.

The multi-edge projection outperformed the simple projection, and scored better than the degree-corrected hypergraph chain. This is particularly interesting in the context of our results on synthetic data, where there appears to be no significant difference between simple and multi-edge projections. One possible explanation is that the multi-edge projection of the synthetic hypergraphs produces a simple graph, without multiple edges, since sparse hypergraphs have few overlapping hyperedges. We caution against drawing a strong conclusion in favor of the multi-edge projection chain over the degree-corrected chain from Fig. 8. In particular, Fig. 8a shows that the error the degree-corrected chain made is in splitting a cluster, which it will not always do. Stronger evidence for using the hypergraph degree-corrected model versus the multi-edge projection is provided by Figs. 5 and 10. For comparison we ran a Scikit-learn^30^ Spectral Clustering implementation on both simple and multi-edge projections (.93 and .91 ARI resp.) and a best of 50 runs Hy-MMSBM (hypergraph mixed-membership stochastic blockmodel)^9^(.171 ARI).

Table 1 reports the model selection procedure for the primary school contact data set; as seen therein, the procedure suggests 6 clusters whereas the ground truth for this data set is 10.

### High school contact hypergraph

The High School Contact data set produced by Mastrandrea et al.^31^ provides a hypergraph with 327 vertices. Edges in this hypergraph correspond to groups of students that were within 1.5 meters of each other and facing each other. The ground truth for this data set assigns students to one of 9 classrooms.

**Figure 9:**
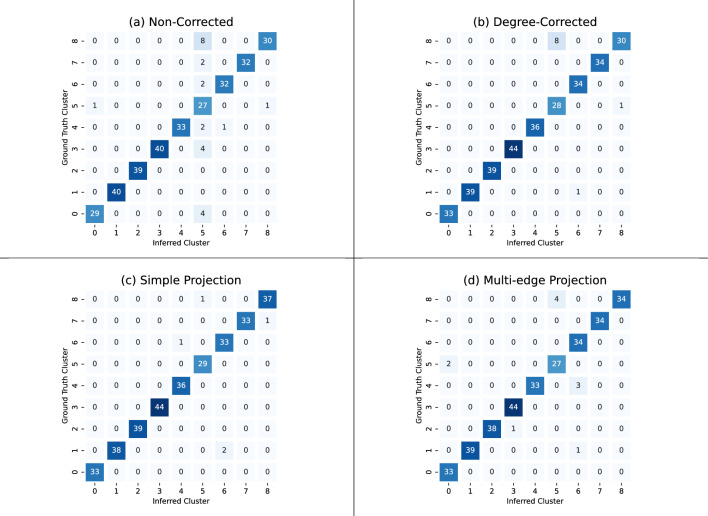
As in Figure 8, using the high-school contact data set^31^. The ARI values are (**a**) 0.84, (**b**) 0.94, (**c**) 0.97, and (**d**) 0.93.

Preliminary exploration of this data set found that the non-corrected chain did not perfectly recover the ground truth clusters. For a representative illustration of the performance of the non-corrected chain, see Fig. 9a. In that particular experiment, we obtained an ARI of 0.84 by selecting the lowest entropy observed across 50 independent runs with 20,000 steps each. This suggests that the non-corrected chain is detecting communities, but that there is room for improvement.

We run the degree-corrected algorithm described in Section “Degree-corrected entropy”, which leads to better community detection. This is illustrated by the cluster heat map (b) in Fig. 9, which achieves an ARI of 0.94.

As with the Primary School Contact Data in Section “Primary school contact hypergraph”, we ran the degree-corrected chain on the simple and multi-edge projections of the data set. The simple projection and multi-edge projection performed comparably with ARIs of 0.97 and 0.93, respectively.

**Figure 10:**
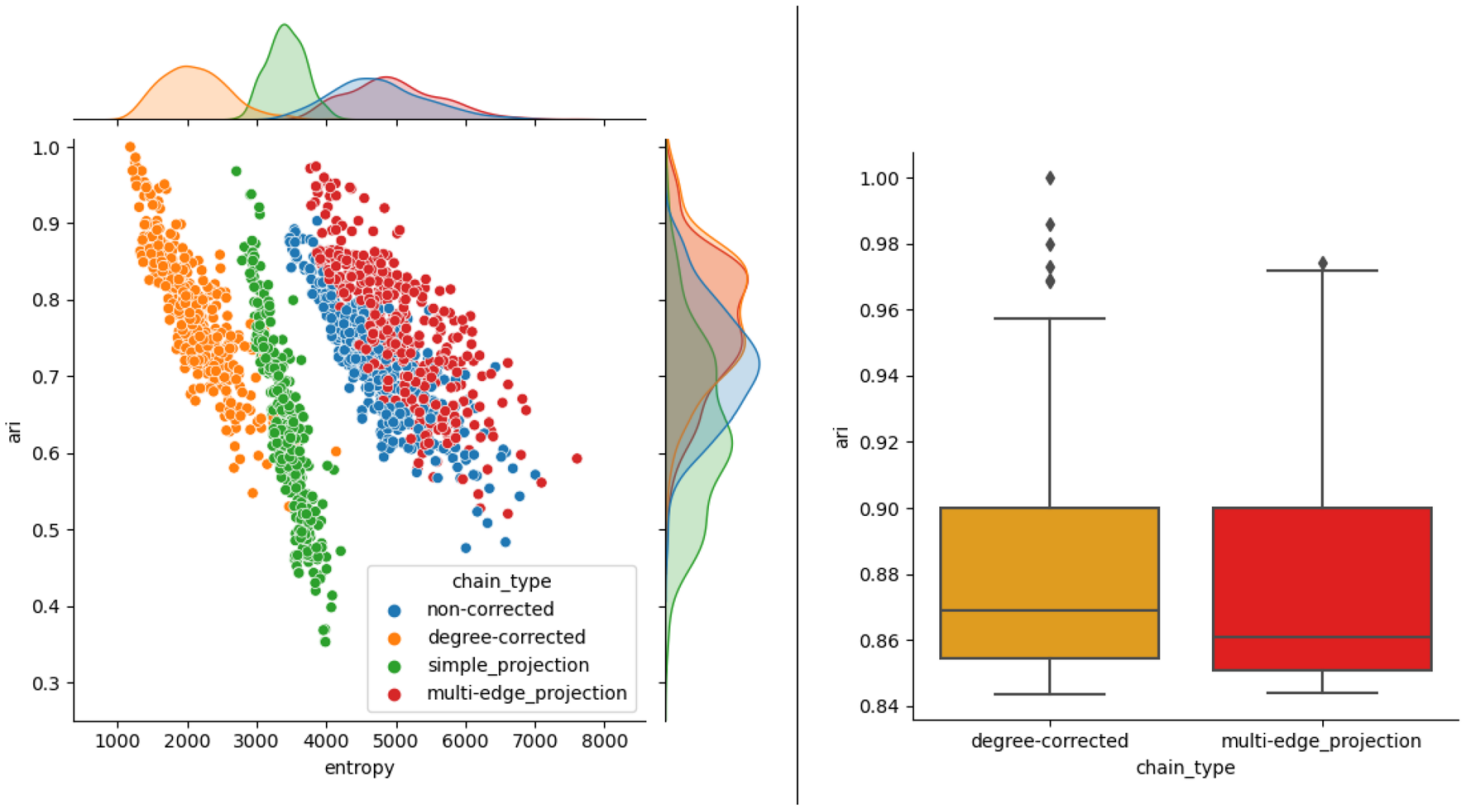
(Left) Each scatter plot consists of 400 points and plots the entropy against the ARI. Each point is obtained by running the corresponding chain for 20,000 steps and keeping the lowest entropy observed on that run. Then, the ARI is calculated using the corresponding clustering. Entropy values are shifted to fall within the interval [0, 10000). We see that the distributions of ARI between degree-corrected and multi-edge clustering are similar, with slight differences occuring in the regime of high ARI. (Right) A box and whisker plot of the top quartile of ARI for degree-corrected and multi-edge clustering. We see that degree-corrected clustering produces the top ARI, is more likely to have exceptional high ARI outcomes, and has a larger mean than the multi-edge clustering.

Though the clusterings in Fig. 9 suggest that the degree-corrected hypergraph and the degree-corrected multi-edge projection chains are comparable, there is evidence to suggest that the degree-corrected chain is better. The scatter plot in Fig. 10 suggests that the degree-corrected hypergraph chain has the best chance of finding the ground truth clustering, as compared to the other chains. Furthermore, the scatter plots show that the entropy of a clustering is inversely correlated with the ARI. Notably, out of the 400 attempts with both the degree-corrected and non-corrected chains, the highest ARI is achieved by the run with the lowest entropy. The box and whisker plot of the top quartile of the degree-corrected hypergraph chain has a higher maximum and mean ARI with more high ARI outliers than the degree-corrected multi-edge projection chain. Since the highest performing ARI runs are of interest, these statistics about the top quartile suggest that an arbitrary run on a degree-corrected hypergraph chain is slightly more likely to yield a better ARI than on a degree-corrected multi-edge projected chain, although the improvement is modest.

For comparison we ran a Scikit-learn^30^ Spectral Clustering implementation on both simple and multi-edge projections (0.95 and 0.981 ARI resp.) and a best of 50 runs Hy-MMSBM (hypergraph mixed-membership stochastic blockmodel)^9^(0.090 ARI).

The model selection results presented in Table 1 suggests 6 clusters for the high school contact data set, whereas the ground truth for this data set is 9.

### Clustering magic: the gathering cards

Magic: the Gathering draft is a trading card game where eight players open randomized packs of cards and take turns picking cards in a hidden draft. After picking 45 cards, players build 23 card decks with which they compete. Cards have associated colors; either black, blue, green, red, white, or any subset thereof (including the empty subset). Due to the mechanics of the game, it is typically extremely disadvantageous to have cards from more than 2 color classes in a deck. This gives players an incentive to draft their cards concentrated around a pair of colors (for example, one player may concentrate on drafting only white, red, and white-red cards).

The Magic: the Gathering drafting community collects data on the outcomes of online drafts and the subsequent games. This data is publicly available through 17Lands.com^32^. In particular, we used the Dominaria United Premier Draft data, which contains the card names (including multiplicity) of all the cards in a player’s card pool after a draft. We ignored the multiplicity to make a hypergraph where the vertex set is the set of all cards that could possibly be drafted, and a hyperedge is a player’s card pool (without multiplicity) after a draft. We ran two experiments with this data.

**Figure 11:**
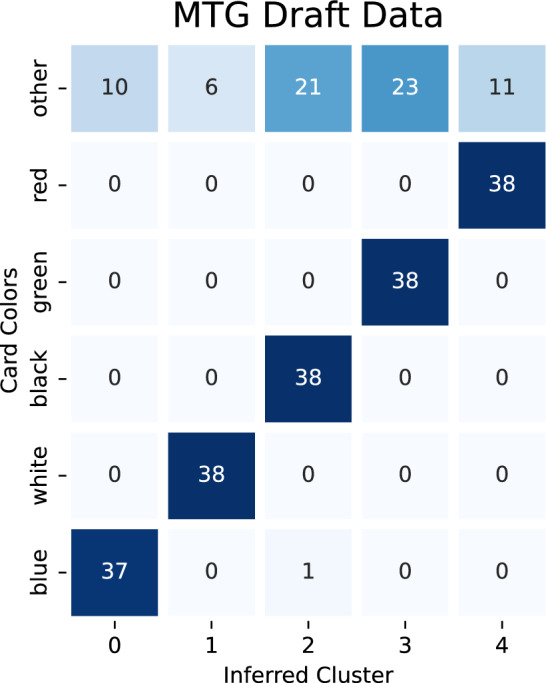
Results from running the degree-corrected chain on the Dominaria United Premier Draft data for 10, 000 steps. The clustering algorithm was looking for 5 clusters, which we break apart into the 5 mono-colored classes and “other” which includes multi-colored and colorless cards.

In the first experiment, we clustered the hypergraph into 5 clusters assuming that a reasonable ground truth would be the colors of the cards. Multi-colored and colorless cards make this notion of ground truth ambiguous. Therefore, we scored the clustering only on how the mono-colored cards are partitioned. The algorithm only mis-classifies a single mono-colored card: the card “Coral Colony” is a blue card that gets clustered with black cards. Results can be found in Fig. 11.

The second experiment applied the minimum description length criterion to determine the number of clusters that are present in the hypergraph. This is motivated by the fact that choosing 8 clusters for the clustering algorithm reveals different deck archetypes. In particular, there are some multi-color strategies that require certain key cards to enable them. Recognizing these archetypes as the “themes” of the clusters requires some domain knowledge, and is therefore, hard to verify independently. However, it does suggest that the minimum description length could reveal a “better” ground truth than card color classes. Unfortunately, our experiment testing different cluster numbers suggests that the 3 clusters provide the shortest description length. This is seen in Table 1 where the suggested number of clusters is 3 on the basis of minimal description length, however, 5 is in some sense the “obvious” number of clusters.

## Discussion

In this document, we establish a novel information-theoretic framework for clustering hypergraph data generalizing the graph theoretic framework established by Rosvall and Bergstrom^10^ while incorporating degree correction methods driven by stochastic blockmodel generative models in the style of Piexoto^11^. We have found that our algorithm is able to recover structures in synthetic and real-world hypergraphs, with performance that is often competitive with methods based on projections of dyadic graphs. Importantly, we find that degree correction leads to modest improvements over non-degree-corrected methods on empirical data sets. We also offer a method based on minimum description-length (MDL) for estimating the appropriate number of communities in data when this is not known *a priori*.

Our results pose several directions of future work. First, our algorithm for clustering is relatively slow. This is due in part to the complicated, highly nonconvex structure of the energy landscape of the entropy minimization objective. Furthermore, our proposed algorithm considers only single-vertex transitions between cluster labels. Merge-split methods such as those discussed by Peixoto (2020) for dyadic graphs may improve performance dramatically^33^. Second, we found further evidence for the established phenomenon of MDL suggesting fewer clusters than ground truth, which warrants further study. Moreover, it would be of considerable interest to empirically benchmark our proposed algorithm in both speed and clustering performance against the many existing hypergraph clustering and partitioning methods in a variety of application areas. Of special interest are algorithms designed for specific domains, such as balanced partitioning^7^, image segmentation^34^, or circuit design^35^ and to theoretically prove consistency as discussed in Section “Results: experiments on data”. Finally, the framework of data analysis as a compression-motivated optimization problem is one which may have use in other directions. Formulating more analysis problems in terms of compression would allow us to deploy combinatorial optimization techniques in the service of complex systems science.

## Data Availability

The data used in Section “Results: experiments on data” consists of the Primary School Contact data and High School Contact data ^31^ as well as the Magic: The Gathering data. ^32^ The first two are available through Austin Benson’s data web page, ^36,37^ while the Magic: The Gathering data is directly available through https://www.17lands.com/. ^32^
